# A new kid in the folding funnel: Molecular chaperone activities of the BRICHOS domain

**DOI:** 10.1002/pro.4645

**Published:** 2023-06-01

**Authors:** Axel Leppert, Helen Poska, Michael Landreh, Axel Abelein, Gefei Chen, Jan Johansson

**Affiliations:** ^1^ Department of Biosciences and Nutrition Karolinska Institutet Huddinge Sweden; ^2^ Department of Microbiology, Tumour and Cell Biology Karolinska Institutet Solna Sweden; ^3^ School of Natural Sciences and Health Tallinn University Tallinn Estonia

**Keywords:** Alzheimer disease treatment, amyloid, blood–brain barrier, molecular chaperone, protein misfolding

## Abstract

The BRICHOS protein superfamily is a diverse group of proteins associated with a wide variety of human diseases, including respiratory distress, COVID‐19, dementia, and cancer. A key characteristic of these proteins—besides their BRICHOS domain present in the ER lumen/extracellular part—is that they harbor an aggregation‐prone region, which the BRICHOS domain is proposed to chaperone during biosynthesis. All so far studied BRICHOS domains modulate the aggregation pathway of various amyloid‐forming substrates, but not all of them can keep denaturing proteins in a folding‐competent state, in a similar manner as small heat shock proteins. Current evidence suggests that the ability to interfere with the aggregation pathways of substrates with entirely different end‐point structures is dictated by BRICHOS quaternary structure as well as specific surface motifs. This review aims to provide an overview of the BRICHOS protein family and a perspective of the diverse molecular chaperone‐like functions of various BRICHOS domains in relation to their structure and conformational plasticity. Furthermore, we speculate about the physiological implication of the diverse molecular chaperone functions and discuss the possibility to use the BRICHOS domain as a blood–brain barrier permeable molecular chaperone treatment of protein aggregation disorders.

## 1 INTRODUCTION

The three‐dimensional structure of proteins and their dynamics are key to their biological function. During their life cycle, proteins encounter various stresses, like heat, oxidative substances, or mechanical damage, which can impair their structure and function. Non‐native protein conformations and aggregates inflict cellular damage, thereby contributing to initiation and progression of several detrimental human disorders, like Alzheimer's disease (AD), Parkinson's disease, and type II diabetes (Aguzzi & O'Connor, 2010; Chiti & Dobson, 2017). For maintaining a healthy proteome, organisms constitutively express a plethora of molecular chaperones that counteract toxic consequences of protein misfolding and aggregation. Especially under stress conditions that increase the concentration of non‐native and aggregation‐prone folding intermediates, the expression of molecular chaperones is upregulated, corroborating their importance for protein homeostasis (Hartl et al., 2011; Kim et al., 2013).

Molecular chaperones can be roughly divided into two groups based on their ability to bind and consume ATP. ATP‐dependent molecular chaperones help substrates adopt their native conformation or prepare them for degradation. ATP‐independent molecular chaperones on the other hand maintain substrates in a folding‐competent state, usually without refolding them, leaving refolding or degradation to other cellular systems (Haslbeck et al., 2005). Therefore, they are often referred to as “holdases,” a paradigm that might need a more precise distinction as recent studies showed that some proteins like DAXX and Spy possess substrate‐specific ATP‐independent chaperone foldase activities (Huang et al., 2021; Mitra et al., 2021). To date, most information regarding ATP‐independent molecular chaperones relates to the small heat shock proteins (sHSPs), which are found mainly intracellularly. However, the number of proteins with ATP‐independent molecular chaperone functions that are being discovered is constantly increasing, emphasizing the omnipresent need for chaperoning inside and outside of the cell. Particularly under conditions or in compartments with low energy supply, this class of molecular chaperones is one of the important housekeepers of a healthy proteome.

Over the past few years, the BRICHOS domains from three proproteins, namely Bri2, Bri3, and proSP‐C, have been established as efficient ATP‐independent molecular chaperones with activities against fibrillar (amyloid) and non‐fibrillar (amorphous) protein aggregation (Chen et al., 2017; Cohen et al., 2015; Poska et al., 2020; Willander, Presto, et al., 2012). The involvement of BRICHOS proproteins in several protein aggregation disorders, particularly connected to amyloid formation, suggests that they are important for the organismal health (Buxbaum & Johansson, 2017; Sanchez‐Pulido et al., 2002). Recent advances concerning the structure, function, and chaperone activities of several BRICHOS domains as well as their apparent applicability in the treatment of amyloid diseases encouraged us to review the current literature. We discuss the structural foundation of their molecular chaperone functions for their “native” (i.e., derived from the same proprotein as the BRICHOS domain) and “non‐native” (i.e., derived from non‐BRICHOS‐containing proteins), aggregation‐prone clients. Finally, the opportunities and challenges of using the BRICHOS domain as a potential treatment for protein aggregation disorders are discussed.

## 2 THE BRICHOS PROTEIN FAMILY

**FIGURE 1:**
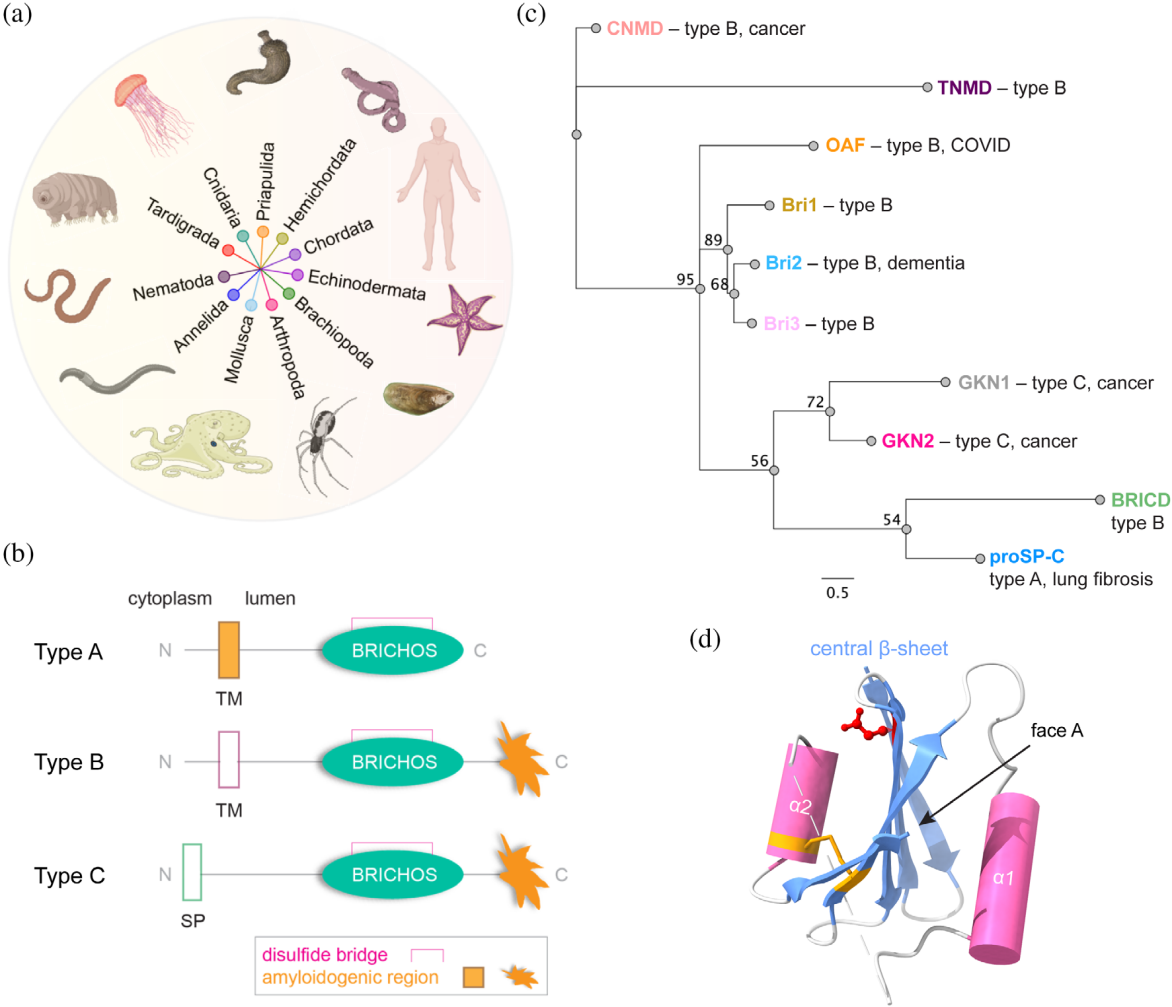
Phylogeny of BRICHOS proproteins and their domain architecture. (a) Phyla of BRICHOS domain‐containing proteins. (b) Common architectures of BRICHOS proproteins with the transmembrane (TM), signal peptide (SP), BRICHOS domain, conserved disulfide, and amyloidogenic region indicated. (c) Neighbor‐joining consensus tree of human BRICHOS families, their architecture, and known disease association. Chondrosarcoma has been associated with CNMD and gastric cancer has been linked to GKN1 and GKN2, while mutations in Bri2 are linked to familial British and Danish dementias (FBD and FDD, respectively). The numerical branch labels correspond to the consensus support (%) and the scale bar refers to a phylogenetic distance of 0.5 substitutions per site. (d) Experimentally solved structure of proSP‐C BRICHOS (PDB 2yad) showing a central β‐sheet flanked by one α‐helix (cylinder) on each side. Face A is assigned as the surface of the central β‐sheet facing α‐helix 1. The conserved disulfide bridge and Asp amino acid residue are heightened in yellow and red, respectively. The dashed line indicates the missing region between the two helices.

Since the past few years, many BRICHOS‐containing protein sequences have been identified due to advances in genome sequence analysis and annotation. The BRICHOS superfamily covers 11 metazoan phyla, including species from worms, fishes to humans (Figure 1a; Chen et al., 2022). In general, BRICHOS proproteins are known or predicted to be type II transmembrane (TM) or secretory proteins that share a similar architecture of an N‐terminal cytosolic domain, a hydrophobic TM region or signal peptide, a linker region, a BRICHOS domain and a C‐terminal domain that has a high β‐strand propensity (Figure 1b). The, so far, only exception is the proSP‐C protein, which lacks the C‐terminal amyloid‐prone region but has an aggregation‐prone TM region instead (Figure 1b; Hedlund et al., 2009; Sanchez‐Pulido et al., 2002). Interestingly, some non‐human genomes code for multiple, consecutive BRICHOS domains along with their corresponding aggregation‐prone regions, but their biological functions are unknown (Chen et al., 2022). Human Bri2, Bri3, and proSP‐C are by far the most studied BRICHOS domain‐containing proproteins and are known to be proteolytically processed along the secretory pathway (Beers et al., 1994; Martin et al., 2009). However, while the BRICHOS domains from Bri2 and proSP‐C are cleaved out from their precursors, and Bri2 BRICHOS is shed into the extracellular space, Bri3 appears to remain membrane‐bound. Other BRICHOS proteins are only sparsely, or not at all, investigated experimentally so far.

To date, >3000 BRICHOS domain‐containing proteins, including species variants, have been identified and based on amino acid sequence alignments, they can be phylogenetically grouped into 14 families, that is, integral membrane protein 2A (ITM2A, also called Bri1), ITM2B (also called Bri2), ITM2C (also called Bri3), group I, group II, gastrokine‐1 (GKN1), gastrokine‐2 (GKN2), gastrokine‐3 (GKN3), tenomodulin (TNMD), chondromodulin (CNMD), proSP‐C, BRICHOS‐containing domain 5 (BRICD5), Out at First (OAF) protein, and antimicrobial peptide (AMP) (Chen et al., 2022; Sanchez‐Pulido & Ponting, 2022). Among these 14 BRICHOS families, 10 families are found in humans (Figure 1c) and several members are associated with severe diseases like cancer, dementia, and COVID‐19 (Hedlund et al., 2009; Sanchez‐Pulido & Ponting, 2022). The physiological functions of the BRICHOS‐containing proproteins are largely unknown, the only exceptions being proSP‐C and Bri2. ProSP‐C is exclusively expressed in alveolar type II cells and multi‐step proteolytic processing of proSP‐C generates SP‐C, which is an integral part of alveolar surfactant that is required for preventing alveolar collapse during expiration, see Johansson and Curstedt (2019) for a review. Bri2 is expressed both in the central nervous system and peripheral organs and has been suggested to take part in neuronal development, synaptic transmission, and regulation of the processing of the amyloid‐β precursor protein, but exact functions remain to be defined, see recent review by Martins et al. (2021).

**FIGURE 2:**
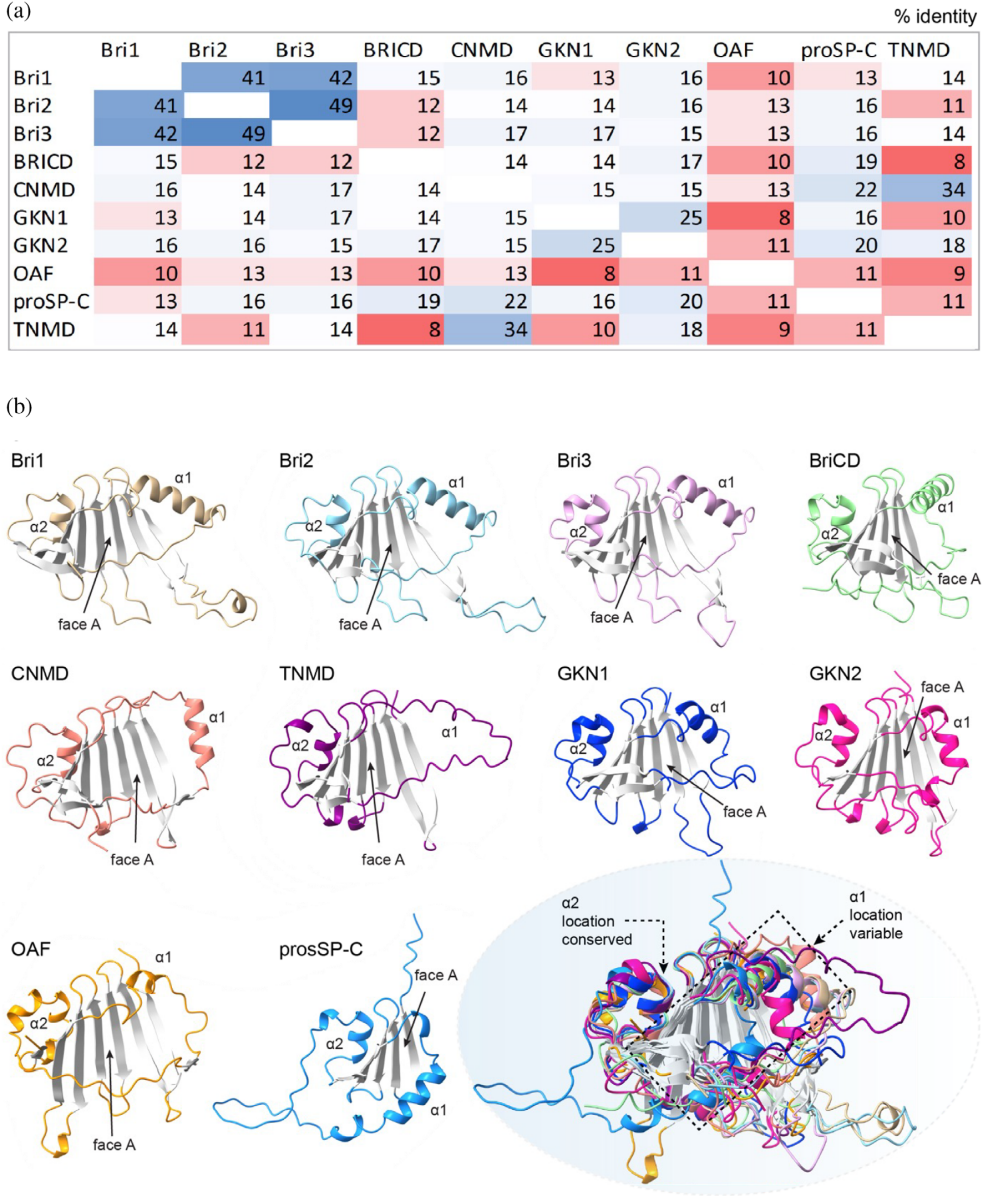
Structure characteristics of all human BRICHOS domains. (a) Pairwise amino acid sequence identities (%) of the 10 human BRICHOS domains color‐coded from low (red) to high (blue) degrees of identities. (b) AF2 structure predictions of human BRICHOS domains from: ITM2A (also known as Bri1, residues 122–227), ITM2B (Bri2, residues 126–231), ITM2C (Bri3, residues 125–230), BRICD5 (residues 89–195), CNMD (residues 97–204), TNMD (residues 86–190), GKN1 (residues 61–166), GKN2 (residues 47–130), OAF (residues 29–130), and proSP‐C (residues 90–197). Bottom right panel shows the overlay of all models where the β‐sheet is colored gray, and the locations of the helices are indicated.

The BRICHOS domains from different families share low pairwise sequence identities (down to <10% between families, Figure 2a) but a strong consensus in their predicted secondary structures, suggesting that the BRICHOS structure is widely preserved (Knight et al., 2013; Sanchez‐Pulido et al., 2002). Therefore, advances in machine learning‐based protein structure prediction algorithms like AlphaFold2 and RosettaFold with homology modeling have already enabled the identification of a new BRICHOS domain‐containing protein (OAF) and may reveal further unknown BRICHOS proteins (Baek et al., 2021; Jumper et al., 2021; Sanchez‐Pulido & Ponting, 2022). The conserved tertiary structure of the BRICHOS domain is characterized by a central β‐sheet that is flanked by two α‐helices (1 and 2; Figure 1d). It is interesting to note that essentially just three residues are highly conserved in the BRICHOS domain, one aspartic acid (Asp) and two cysteine (Cys) residues (Figure 1b; Hedlund et al., 2009). The Cys residues form an intramolecular disulfide bond that connects helix 2 and the central β‐sheet in proSP‐C BRICHOS, Bri2 BRICHOS, and likely all other BRICHOS domains, reflected by the conserved location of helix 2 within human BRICHOS domains (Figure 2b; Chen et al., 2017; Willander, Askarieh, et al., 2012). In contrast, the location of helix 1 is less well‐defined in structure predictions and likely more flexible in most human BRICHOS domains (Figure 2b). The flexible and dynamic nature of helix 1 might be important for BRICHOS' function, enabling the exposure of substrate binding sites. The only exception is human proSP‐C BRICHOS which has two additional Cys that form an intramolecular disulfide bond between the loop region just after helix 1 and face A, but this disulfide is not conserved within the BRICHOS family. The strictly conserved Asp and Cys residues have been studied in Bri2 and proSP‐C BRICHOS and found to be important for generating and regulating different BRICHOS molecular chaperone functions (Chen et al., 2022; Leppert et al., 2022).

## 3 BRICHOS STRUCTURE AND NATIVE CLIENT RECOGNITION

**FIGURE 3:**
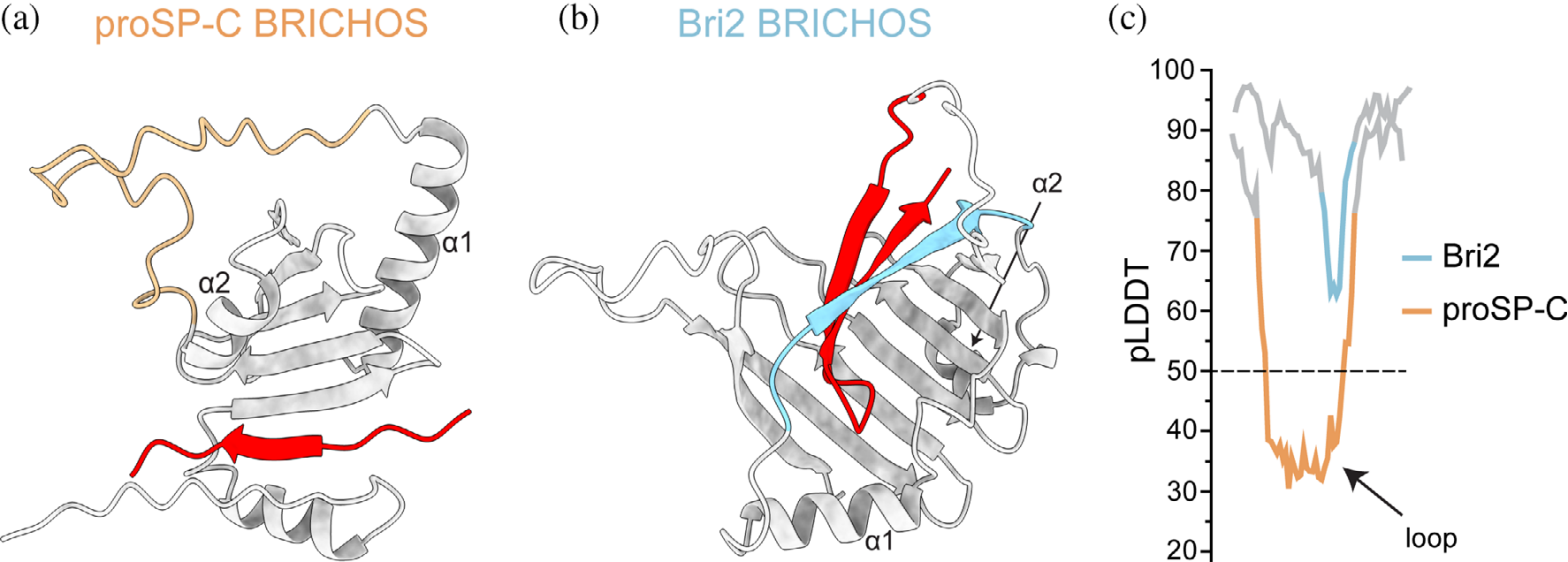
Native client binding of BRICHOS domains. AF2 predictions of (a) human proSP‐C BRICHOS (residues 60–197; gray and yellow) in complex with a poly‐Val peptide (red) (adopted from Osterlund et al. (2022)) and (b) Bri2 BRICHOS domain (gray and blue) and C‐terminal aggregation‐prone region (Bri23, red; residues 85–266). (c) Per‐residue confidence score (pLDDT) plots around the region connecting helix 1 and 2 in proSP‐C BRICHOS (in panel (a), residues 140–197) and Bri2 BRICHOS (in panel (b), residues 160–220).

The two most studied BRICHOS domains in terms of structural properties are proSP‐C BRICHOS and Bri2 BRICHOS. The only high‐resolution structure to atomic detail of a BRICHOS domain is derived from x‐ray crystallography data of human proSP‐C BRICHOS demonstrating the conserved BRICHOS fold (Figure 1d; Willander, Askarieh, et al., 2012). However, a long stretch, which is not resolved in the crystal structure connects both helices. The AF2 model of the folded proSP‐C BRICHOS core superimposes very well with the experimental structure and suggests that both helices are connected by an unstructured loop, indicated by a low per‐residue confidence score (pLDDT << 50; Figure 3a,c). Initially, molecular dynamic (MD) simulations of proSP‐C BRICHOS suggested that movement of helix 1 exposes face A, which in general has residues with side‐chain physicochemical properties that are complementary to the properties of the amyloidogenic region of the corresponding BRICHOS proprotein, and thus likely serves as a client binding region (Fitzen et al., 2009; Knight et al., 2013; Willander, Askarieh, et al., 2012). The dynamic nature of helix 1 may hence be an important feature to expose face A for client binding. However, recent data suggest that also an alternative possibility for the client—proSP‐C BRICHOS interaction may exist by which a β‐strand prone poly‐Val peptide sequence, resembling features of the native client SP‐C, is trapped in a groove formed by a complementary β‐strand of the N‐terminal linker and the first β‐strand of the BRICHOS domain (Figure 3a). This linker is a frequent target of disease‐associated mutations, and this binding mode could thus explain the loss of chaperoning capacity of the proSP‐C BRICHOS domain for its native client as a result of mutations (Osterlund et al., 2022; Willander, Askarieh, et al., 2012).

AF2‐based structural models show that the loop connecting helix 1 and 2, which is unstructured in proSP‐C BRICHOS (low pLDDT), adopts a β‐strand conformation (high pLDDT score) in most other human BRICHOS domains, in line with previous secondary structure predictions (Hedlund et al., 2009). In these cases, this β‐strand together with residues in face A stabilizes the C‐terminal aggregation‐prone region of the respective BRICHOS protein in a β‐hairpin conformation (Figure 3b). These observations suggest a distinct client recognition mechanism of the proSP‐C BRICHOS domain, the only BRICHOS protein with its native client located N‐terminally of the BRICHOS domain. The mechanism of complementary β‐strand trapping might, however, be a common feature within the BRICHOS family.

## 4 STRUCTURAL PLASTICITY OF THE BRICHOS DOMAIN

**FIGURE 4:**
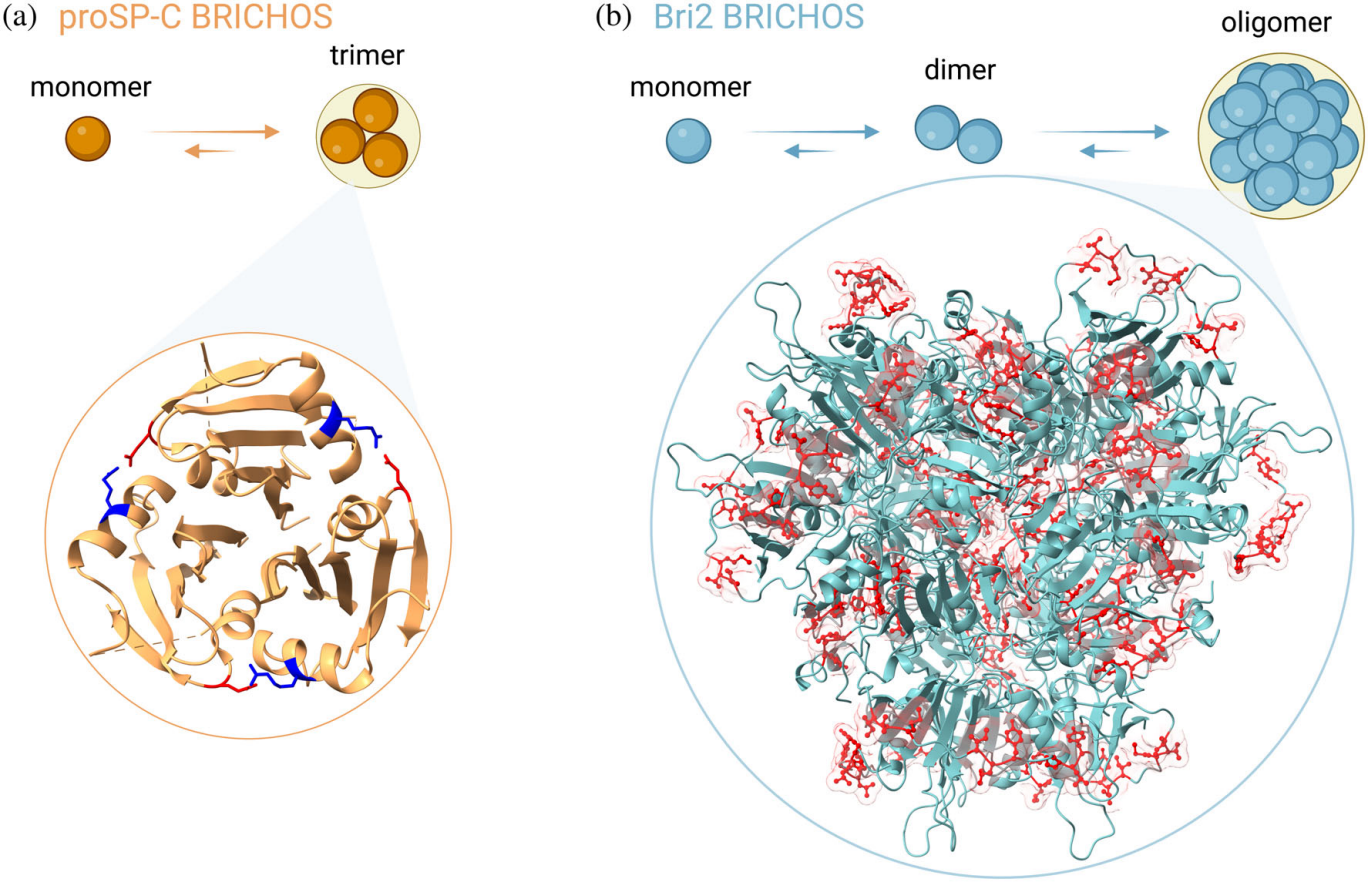
Structures of BRICHOS assemblies. (a) proSP‐C BRICHOS trimer (PDB 2yad) with the salt bridges highlighted (blue and red). (b) Cryo‐EM structural model of the Bri2 BRICHOS oligomer, with 24 subunits, where hydrophobic residues in the loop region (red) are highlighted.

In vitro, proSP‐C BRICHOS exists as an equilibrium between monomers and mainly homotrimers that are stabilized by a salt bridge and other non‐covalent interactions (Figure 4a; Biverstål et al., 2015; Willander, Askarieh, et al., 2012). The non‐covalently linked proSP‐C BRICHOS trimers can be fully dissociated by detergents or point mutations that interfere with the interface (Leppert et al., 2021). Notably, native mass spectrometry experiments have shown that proSP‐C BRICHOS—polyV (polyVal is a sequence feature of the native client SP‐C) interactions depend on the monomer conformation, as formation of the binding cleft is blocked in the trimer (Osterlund et al., 2022). In contrast to proSP‐C BRICHOS, Bri2 BRICHOS, and Bri3 BRICHOS exist as monomers, dimers, and n‐mers of dimers up to polydisperse high molecular weight assemblies (Figure 4b). Bri2 BRICHOS assemblies are stabilized by non‐covalent interactions as well as a variable number of disulfide bridges (Chen et al., 2017; Leppert et al., 2022). The assembly of Bri2 BRICHOS oligomers from monomers is promoted by reducing conditions through cycles of reduction and re‐oxidation of intra‐ and intermolecular bonds with distinct thiol reactivities (Leppert et al., 2022). The assembly process leads to an increase of the overall surface hydrophobicity and exposure of short hydrophobic motifs from the Bri2 BRICHOS loop. The physiological relevance of this mechanism remains to be established, but we speculate that in an overall more oxidative environment (e.g., the extracellular space), BRICHOS molecular chaperone functions could be generated upon reductive stress insults. A cryo‐EM structural model of Bri2 BRICHOS oligomers shows particles consisting of 24 subunits with a tetrahedral symmetry, where many of the loop regions between helix 1 and 2 are solvent exposed (Figure 4b). Many classical sHSPs form large polydisperse oligomers with dimers as the basic building block (Basha et al., 2012, 2013; Haslbeck & Vierling, 2015; Santhanagopalan et al., 2018). But in contrast to the disulfide‐dependent dimer association and oligomer assembly of Bri2 BRICHOS, sHSPs oligomer assembly depends on non‐covalent interactions involving multiple regions of the subunits.

## 5 MOLECULAR CHAPERONE FUNCTIONS FOR NON‐NATIVE CLIENTS

Molecular chaperones bind a wide variety of substrates to prevent their unwanted aggregation and associated toxic consequences. Protein misfolding can result in non‐fibrillar (amorphous) or fibrillar (amyloid) aggregation with characteristic and widely different end‐point structures. Amorphous protein aggregation is the assembly of misfolded proteins in an insoluble structure mediated by fuzzy intermolecular contacts often via exposed hydrophobic stretches (Song, 2009). These proteins or aggregates can harm an organism due to loss of protein function or gain of toxic activity. In contrast to amorphous aggregates, amyloid fibrils are highly structured self‐assemblies with an ordered cross‐β structure (Eisenberg & Jucker, 2012). The mechanisms and kinetics of nucleation‐dependent amyloid fibril formation have been extensively investigated and can be described by three nucleation steps: (1) Primary nucleation, that is the association of monomeric peptides into oligomers, that eventually convert into fibrils, (2) elongation, that is the process of adding monomers to fibril ends, and (3) secondary nucleation, that is the catalytic formation of oligomers on the surface of already existing fibrils (Arosio et al., 2015; Cohen et al., 2013; Knowles et al., 2009). Concerning the AD‐associated 42‐residue amyloid‐β (Aβ42) peptide, it appears that oligomers are the main toxic species that also correlate best with AD pathology (De et al., 2019; Tomic et al., 2009; Wang et al., 2019; Yang et al., 2017). The interference with specific nucleation steps in the amyloid aggregation pathway by molecular chaperones has different effects on the generation of toxic aggregates. In case of Aβ42, blocking secondary nucleation greatly reduces the formation of oligomers, while preventing fibril end elongation increases the number of toxic oligomers (Arosio et al., 2016; Cohen et al., 2015; Linse et al., 2020). Therefore, it is important to understand the effects of anti‐amyloid drugs and molecular chaperones to molecular detail.

Interestingly, the Bri2 and proSP‐C BRICHOS domains interfere also with the aggregation pathways of other amyloidogenic peptides than their native clients, like Aβ, islet amyloid polypeptide, α‐synuclein, β17, and medin (Biverstal et al., 2020; Johansson, Nerelius, et al., 2009; Nerelius et al., 2009; Oskarsson et al., 2018; Sarr et al., 2018). In vitro, the proSP‐C BRICHOS domain mainly prevents surface‐catalyzed secondary nucleation of Aβ42 and associated cytotoxicity, has only small effects on fibril‐end elongation and does not interact with Aβ42 monomers (Cohen et al., 2015; Leppert et al., 2021). Interestingly, these functions are independent of the assembly state of the proSP‐C BRICHOS domain (i.e., monomer or trimer), suggesting different binding modes for Aβ42 fibrils and SP‐C (Biverstål et al., 2015; Cohen et al., 2015; Leppert et al., 2021). The ability of the proSP‐C BRICHOS domain to specifically affect a single nucleation rate is not shared by the BRICHOS domains from Bri2 and Bri3, which significantly affect fibril‐end‐elongation in addition to secondary nucleation (Chen et al., 2017; Poska et al., 2020). These functions are, like for proSP‐C BRICHOS, not linked to one assembly state of the BRICHOS domain; however, there are important differences in their abilities to affect Aβ42 kinetics and Aβ42‐associated neurotoxicity. It appears that Bri2 BRICHOS dimers are most efficient in delaying amyloid fibrillation, but monomers reduce Aβ42‐induced neurotoxicity to a greater extent. These differences might be explained by the ability of dimers to bind better to fibril ends, while the double effective number of monomers compared to dimers may more efficiently cover the surface of fibrils and thereby predominantly prevent the formation of toxic Aβ42 oligomers (Chen et al., 2017, 2020).

The inability of proSP‐C BRICHOS and Bri2 BRICHOS to bind Aβ monomers is somewhat surprising given that Aβ, like SP‐C and Bri23 (the native clients of proSP‐C and Bri2 BRICHOS, respectively, see Figure 3), has hydrophobic stretches with high β‐strand propensities (Kallberg et al., 2001). These observations lead us to speculate that binding of the isolated BRICHOS domain requires proximity of several binding sites provided in fibrils, and/or that Aβ monomers exist in a BRICHOS domain incompatible conformation in solution. Fluorescence correlation spectroscopy indicated that proSP‐C BRICHOS can bind Aβ42 oligomers composed of eight or less molecules (Leppert et al., 2021). The only other BRICHOS domains that so far have been studied concerning their anti‐amyloidogenic properties are from human Bri3 and human gastrokine 1 (Altieri et al., 2014; Poska et al., 2020).

While all BRICHOS domains studied to date show anti‐amyloid activities only the BRICHOS domains from Bri2 and Bri3 prevent amorphous, non‐fibrillar protein aggregation by forming transient complexes with misfolded clients (Chen et al., 2017, 2022; Poska et al., 2016, 2020). This feature is linked to the exposure of substrate binding sites made up of short hydrophobic motifs that are brought together via disulfide‐dependent oligomerization processes (Chen, Leppert, et al., 2023). The mechanism of substrate binding of BRICHOS oligomers is appealingly similar to ATP‐independent molecular chaperones, like sHSP26 and sHSP42 from baker's yeast, which bind non‐selectively to misfolded substrates (Haslbeck & Vierling, 2015; Poska et al., 2016). However, the gain of chaperone function via a disulfide‐dependent assembly mechanism seems to be unique for the Bri2 BRICHOS domain.

## 6 CHAPERONE‐LIKE ACTIVITIES OF BRICHOS AT TWO DIFFERENT LEVELS AND RELATIONS TO HUMAN DISEASE

The emerging picture is that the BRICHOS domain is a versatile building block that is used in molecular chaperone activities against protein misfolding and aggregation at two levels.

First, the BRICHOS domain, as a constituent of proproteins, can prevent misfolding of specific and highly amyloidogenic regions (Figure 1b). This is proposed to occur during biosynthesis and this function of the BRICHOS domain is based on data for the proSP‐C protein that stabilizes and promotes correct folding of its aggregation‐prone TM segment (Johansson et al., 2006; Johansson, Eriksson, et al., 2009; Nerelius et al., 2008; Pobre‐Piza et al., 2022). Recombinant BRICHOS domain from proSP‐C in isolation has been shown to prevent SP‐C (the amyloidogenic part of proSP‐C) from forming amyloid fibrils (Nerelius et al., 2008). Data suggest that the BRICHOS domain binds in trans to the aggregation‐prone region of a nearby molecule and thereby affords a scaffold for correct folding and avoidance of β‐sheet aggregation (Pobre‐Piza et al., 2022). Several mutations in the proSP‐C gene are linked to familial interstitial lung disease (ILD) with lung fibrosis, many of those mutations are located to the linker and BRICHOS domain, and apparently, they are not found in healthy individuals, which strongly suggest that they are linked to development of the disease state (Beers & Mulugeta, 2005; Gustafsson et al., 1999; Pobre‐Piza et al., 2022; Willander, Askarieh, et al., 2012). The development of ILD and amyloid in childhood in association with proSP‐C BRICHOS mutations points to the strongly amyloidogenic nature of poly‐Val in SP‐C and the importance of BRICHOS, considering that other amyloid diseases generally occur late in life. Moreover, modeling some of these mutations in vitro and in animals’ support that they result in protein misfolding, recruitment of molecular chaperones, cellular stress, and fibrotic remodeling (Nureki et al., 2018; Pobre‐Piza et al., 2022; Saenz et al., 2015). In case of Bri2, three different mutations in the ITM2B gene lead to the release of extended C‐terminal peptides that deposit primarily in the central nervous system as amyloid (Liu et al., 2021; Mead et al., 2000; Vidal et al., 1999, 2000). These peptides, referred to as ABri and ADan, have been found causative of familial British and familial Danish dementia (FBD and FDD), respectively, that share clinical and pathological similarities with AD. However, for all BRICHOS‐associated misfolding diseases, the exact molecular mechanisms regarding the BRICHOS domain remain to be defined.

Second, the BRICHOS domains from proSP‐C, Bri2, and Bri3 in isolation, that is, in the absence of the rest of the corresponding proproteins, inhibit fibrillar and non‐fibrillar aggregation of several proteins and peptides. Intriguingly, the activity to inhibit amyloid fibril formation has been in the focus in several studies from ex vivo in hippocampal slice preparations to in vivo in *Drosophila melanogaster* and mouse models (Chen et al., 2017; Cohen et al., 2015; Dolfe et al., 2018; Hermansson et al., 2014; Manchanda et al., 2023; Poska et al., 2016). The emerging picture is that the BRICHOS domain delays amyloid fibril formation in particular of Aβ42 via apparently unique mechanisms. In this regard, proSP‐C BRICHOS and Bri2 BRICHOS have been demonstrated by fibril formation kinetic experiments to mainly reduce the catalytic formation of new, toxic oligomeric species from Aβ42 monomers on the surface of fibrillar or prefibrillar aggregates (Cohen et al., 2015; Poska et al., 2016). This effect has also been experimentally verified, as the BRICHOS domain reduces Aβ42 mediated neurotoxicity to mouse hippocampal slice preparations and extends the longevity and locomotor activity of a BRICHOS overexpressing fly model. Notably, the degrees of treatment effects of Bri2 BRICHOS in Alzheimer mouse models correlate surprisingly well with predictions of degrees of reduction of toxic oligomers made from in vitro data (Abelein & Johansson, 2023; Manchanda et al., 2023). Misfolding diseases are often related to old age coinciding with a decline of the proteostasis capacity, and further research is required to understand the function of isolated BRICHOS domains under physiological conditions and in relation to human disease development.

## 7 BRICHOS AS A TREATMENT FOR PROTEIN AGGREGATION DISORDERS

Considering that there are currently more than 40 amyloidogenic peptides associated with devastating amyloid diseases, there is a great need for new treatment strategies (Buxbaum et al., 2022).

The abilities of the BRICHOS domain to selectively block surface catalyzed secondary nucleation of Aβ and to prevent the formation and toxicity of formed aggregates, as demonstrated by kinetic analyses of Aβ fibril formation (Arosio et al., 2016; Cohen et al., 2015), effects on hippocampal neuronal network activity (Andrade‐Talavera et al., 2021; Cohen et al., 2015; Poska et al., 2016) and correlations between BRICHOS effects in vivo and in vitro (Abelein & Johansson, 2023), are apparently unique to all so far studied chaperone types. The facts that the Bri2 protein is naturally present in the brain and linked to brain‐localized amyloid diseases make the BRICHOS domain of Bri2 a relevant candidate for a possible disease‐modifying agent in AD.

**FIGURE 5:**
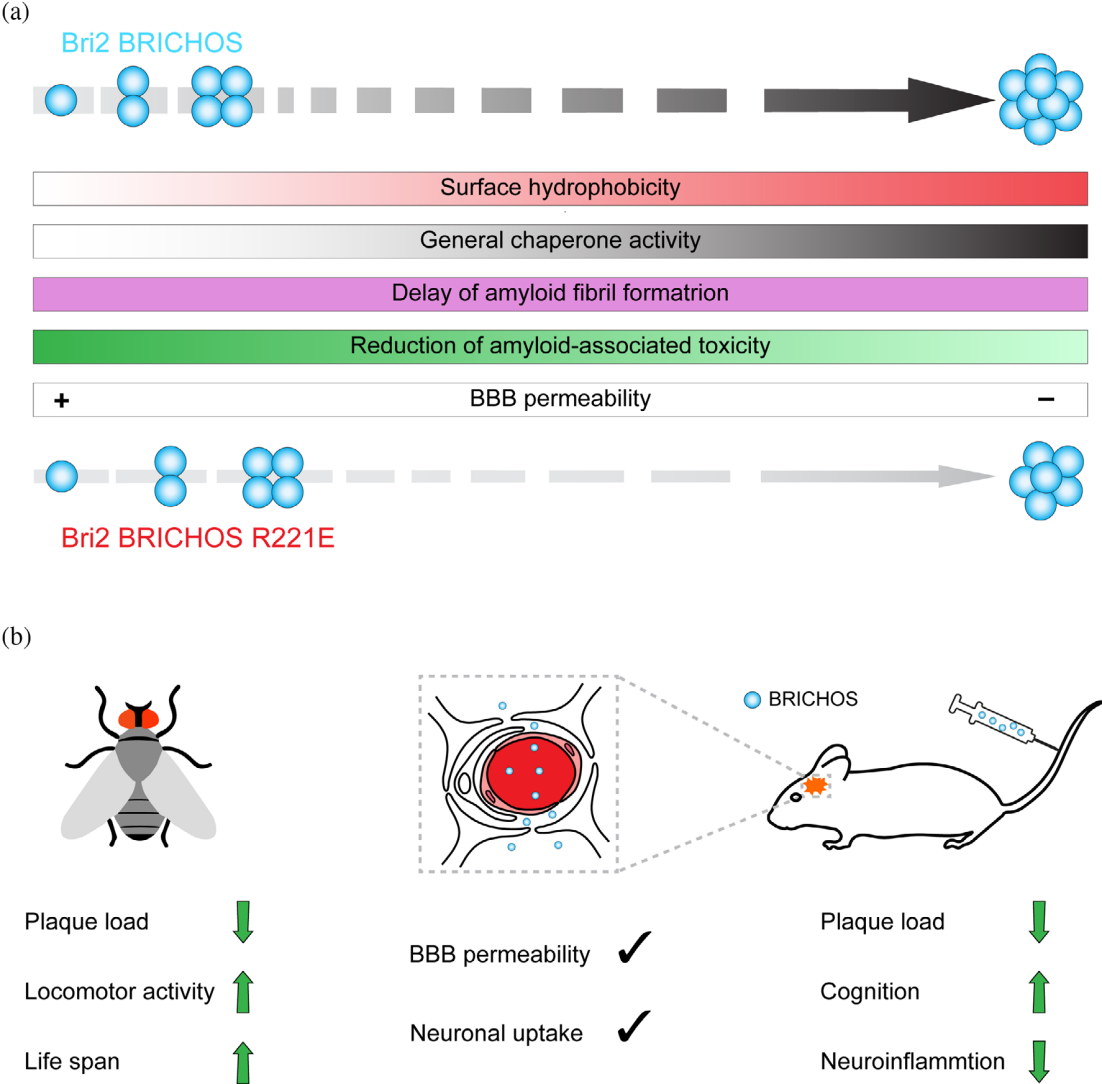
Grammar of the BRICHOS molecular chaperone functions and treatment potential. (a) BRICHOS molecular chaperone functions and blood–brain barrier permeability depends on the assembly state which can be modulated by targeted mutagenesis. (b) BRICHOS can cross the blood–brain barrier and overexpression or intravenous administration in an AD model showed positive effects. See text for details. AD, Alzheimer's disease.

Many chaperones can prevent the formation and toxicity of amyloid aggregates, but no medical treatments have so far been developed based on these features. The possibility of treating neurodegenerative disease with recombinant human Bri2 BRICHOS domain is gaining attraction by the fact that intravenously administered recombinant BRICHOS domain from Bri2, but not proSP‐C, can cross the blood–brain barrier (BBB) in mice and that recombinant Bri2 BRICHOS apparently accumulates in brain parenchyma and neurons after repeated injections (Galan‐Acosta et al., 2020; Manchanda et al., 2023; Tambaro et al., 2019; see Figure 5). To what extent these findings are transferrable to humans remain to be determined. Furthermore, recombinantly produced Bri2 BRICHOS can be internalized by a subset of neuronal cells in hippocampus and cortex, the identities of which remain to be determined (Galan‐Acosta et al., 2020). The mutant R221E that stabilizes the Bri2 BRICHOS monomer conformation can be produced in large scale and recently showed positive outcomes regarding memory functions and astro‐ and microgliosis using AD‐like human Aβ precursor protein (APP) knock in mouse models (Manchanda et al., 2023; Schmuck et al., 2021). Recent ex vivo hippocampal slice experiments suggest that also already impaired neural network oscillations may be restored using the BRICHOS domain (Andrade‐Talavera et al., 2021). Administration of Bri2 or proSP‐C BRICHOS domains to the CNS via an adeno‐associated virus vector injected at birth in a transgenic APP AD mouse model resulted in improved memory and reduced astrogliosis in both cases, further supporting the relevance of the apparently generic ability of BRICHOS domains to reduce Aβ42 neurotoxicity (Dolfe, 2016). Delivery of proteins to the CNS for treatment of neurological diseases remains a major hurdle due to the restrictive nature of the BBB. Delivery of protein drugs might be achieved by targeted focused ultrasound (FUS) which has been proven to be a safe non‐invasive method in preclinical applications for transiently opening the BBB (Sierra et al., 2017). The BRICHOS domains from proSP‐C and Bri2 in combination with FUS and tail vein injected lipid microbubbles resulted in increased brain uptake, but only Bri2 BRICHOS was spread outside the FUS targeted area (Galan‐Acosta et al., 2020). These results together with the ability of Bri2 BRICHOS to pass the BBB in mice after intravenous injections suggest that it is transported into the CNS by a mechanism that should be studied further (Manchanda et al., 2023; Tambaro et al., 2019).

## 8 CONCLUSIONS

The BRICHOS domain has been shown to be an efficient molecular chaperone domain with activities against different types of protein aggregation depending mainly on assembly state and surface hydrophobicity, and has a unique ability among chaperones to pass the BBB (Figure 5). These features and potential manipulations of the biochemical properties make the BRICHOS domain interesting for studies of protein folding, misfolding, and toxicity, as well as for drug development against diseases associated with protein misfolding.
